# The Paradox of Astroglial Ca^2 +^ Signals at the Interface of Excitation and Inhibition

**DOI:** 10.3389/fncel.2020.609947

**Published:** 2020-11-26

**Authors:** Laura C. Caudal, Davide Gobbo, Anja Scheller, Frank Kirchhoff

**Affiliations:** Department of Molecular Physiology, Center for Integrative Physiology and Molecular Medicine, University of Saarland, Homburg, Germany

**Keywords:** astrocyte, Ca^2+^, glutamate, γ-aminobutyric acid, epilepsy, gliotransmission, network plasticity

## Abstract

Astroglial networks constitute a non-neuronal communication system in the brain and are acknowledged modulators of synaptic plasticity. A sophisticated set of transmitter receptors in combination with distinct secretion mechanisms enables astrocytes to sense and modulate synaptic transmission. This integrative function evolved around intracellular Ca^2+^ signals, by and large considered as the main indicator of astrocyte activity. Regular brain physiology meticulously relies on the constant reciprocity of excitation and inhibition (E/I). Astrocytes are metabolically, physically, and functionally associated to the E/I convergence. Metabolically, astrocytes provide glutamine, the precursor of both major neurotransmitters governing E/I in the central nervous system (CNS): glutamate and γ-aminobutyric acid (GABA). Perisynaptic astroglial processes are structurally and functionally associated with the respective circuits throughout the CNS. Astonishingly, in astrocytes, glutamatergic as well as GABAergic inputs elicit similar rises in intracellular Ca^2+^ that in turn can trigger the release of glutamate and GABA as well. Paradoxically, as gliotransmitters, these two molecules can thus strengthen, weaken or even reverse the input signal. Therefore, the net impact on neuronal network function is often convoluted and cannot be simply predicted by the nature of the stimulus itself. In this review, we highlight the ambiguity of astrocytes on discriminating and affecting synaptic activity in physiological and pathological state. Indeed, aberrant astroglial Ca^2+^ signaling is a key aspect of pathological conditions exhibiting compromised network excitability, such as epilepsy. Here, we gather recent evidence on the complexity of astroglial Ca^2+^ signals in health and disease, challenging the traditional, neuro-centric concept of segregating E/I, in favor of a non-binary, mutually dependent perspective on glutamatergic and GABAergic transmission.

## Introduction

The path that led the scientific community to agree upon the role of astrocytes in actively tuning and modulating brain activity has been one of the most challenging and fertile fields in neuroscience for the last decades. It is now widely accepted that astrocytes can sense, react to and modify the extracellular transmitter *milieu* both quantitatively and qualitatively, thus contributing to neural network excitability and functioning. Nevertheless, little is still known about the exact molecular mechanisms of the astrocytic response and contribution to synaptic transmission. Most studies have been characterizing astrocytes in terms of their inputs and outputs, without precise knowledge of their inner working, thus regarding them as *black boxes.* Nowadays, internal Ca^2+^ oscillations are by far considered the main read-out of astrocytic activity (Bazargani and Attwell, 2016) and are known to be induced, among others, by binding of neurotransmitters to astroglial membrane receptors and to eventually lead to the release of gliotransmitters in the extracellular space. These include glutamate (Parpura et al., 1994; Pasti et al., 1997; Kang et al., 1998; Parri et al., 2001; Angulo et al., 2004; Fellin et al., 2004), ATP (Pascual et al., 2005; Serrano et al., 2006), D-serine (Henneberger et al., 2010) and γ-aminobutyric acid (GABA; Liu et al., 2000; Kozlov et al., 2006; Lee et al., 2010; Jiménez-González et al., 2011). In this review we point out that a pile of evidence is building up against a simplistic way of considering astrocytic Ca^2+^ response as a linear and stereotypical process. In order to understand the brain, it is essential to regard astrocytes as active information integrators and processors.

## Astroglial Ca^2+^ Dynamics at the Interface of Glutamatergic and Gabaergic Signaling

The astroglial role as mere responders to neuronal firing was challenged by the fact that astrocytes exhibited internal Ca^2+^ oscillations in hippocampal slice preparations even in presence of the neuronal voltage-gated Na^+^ channel blocker tetrodotoxin (Nett et al., 2002). This confirmed previous *in vitro* evidence of neuron-independent Ca^2+^ activity (Araque et al., 1999; Parri et al., 2001). These results were then similarly obtained using genetically encoded Ca^2+^ indicators (GECIs; Haustein et al., 2014; Bindocci et al., 2017). Several lines of evidence suggested that the spontaneous opening of a member of the transient receptor potential (TRP) family, TRPA1, and possibly of other cation channels of the same family, contribute to resting astroglial Ca^2+^ levels and at least a fraction of their intrinsic fluctuations (Shigetomi et al., 2011, 2013; Agarwal et al., 2017). However, neither specific TRPA1 mRNA or protein was so far detected in astrocytes (Verkhratsky et al., 2014). In line with these observations, 0 mM [Ca^2+^]_o_ reduced the Ca^2+^ transient frequency of the gliapil by up to 75% and the knockout of the inositol triphosphate type 2 receptor (IP3R2) spared around 10% of somatic and around 40% of gliapil fluctuations without affecting the frequency of the latter (Srinivasan et al., 2015). Since tetrodotoxin prevents action potential generation and not neurotransmission itself, these results can, at least partially, be attributed to astroglial responses elicited by spontaneous neurotransmitter release, as shown for the activation of cortical astrocytes by glutamate and ATP (Palygin et al., 2010; Lalo et al., 2011).

Indeed, astroglial Ca^2+^ oscillations driven by extracellular inputs superimpose on and integrate intrinsic Ca^2+^ activity, thus making astrocytes active partners of network functioning. With some notable exceptions (Jennings et al., 2017; Xin et al., 2019), activation of G-protein coupled receptors (GPCRs) leads to intracellular Ca^2+^ elevations (Kofuji and Araque, 2020) not only upon stimulation with molecules commonly considered excitatory, such as glutamate, but also with the canonical inhibitory neurotransmitter GABA (Perea et al., 2016; Mariotti et al., 2018; Mederos and Perea, 2019; Nagai et al., 2019). In line with these observations and contrary to their neuronal counterpart (Huang and Thathiah, 2015), *in vivo* chemogenetic activation of both G_q_ and G_i/o_ DREADDs elicited Ca^2+^ increases in astrocytes (Durkee et al., 2019), thus challenging the long-established concept of E/I as mutually interplaying and yet still discernable processes (Isaacson and Scanziani, 2011). [Ca^2+^]_i_ increase can lead to the release of glutamate (Parpura et al., 1994; Pasti et al., 1997; Kang et al., 1998; Parri et al., 2001; Angulo et al., 2004; Fellin et al., 2004) as well as of GABA (Liu et al., 2000; Kozlov et al., 2006; Lee et al., 2010; Jiménez-González et al., 2011). Notably, contrary to Ca^2+^ uncaging or inositol-1,4,5-trisphosphate (IP3) application, G_q_-coupled receptor activation does not necessarily induce the release of gliotransmitters (Wang et al., 2013) and different G_q_-coupled receptors can exert gliotransmitter release with different efficiencies (Shigetomi et al., 2008). This challenges the idea that astrocytes may act as a redundant layer that responds similarly to GPCR-mediated inputs (Guerra-Gomes et al., 2017) and suggests that astrocytes can discriminate metabotropic signaling upstream of internal Ca^2+^ oscillations.

**FIGURE 1:**
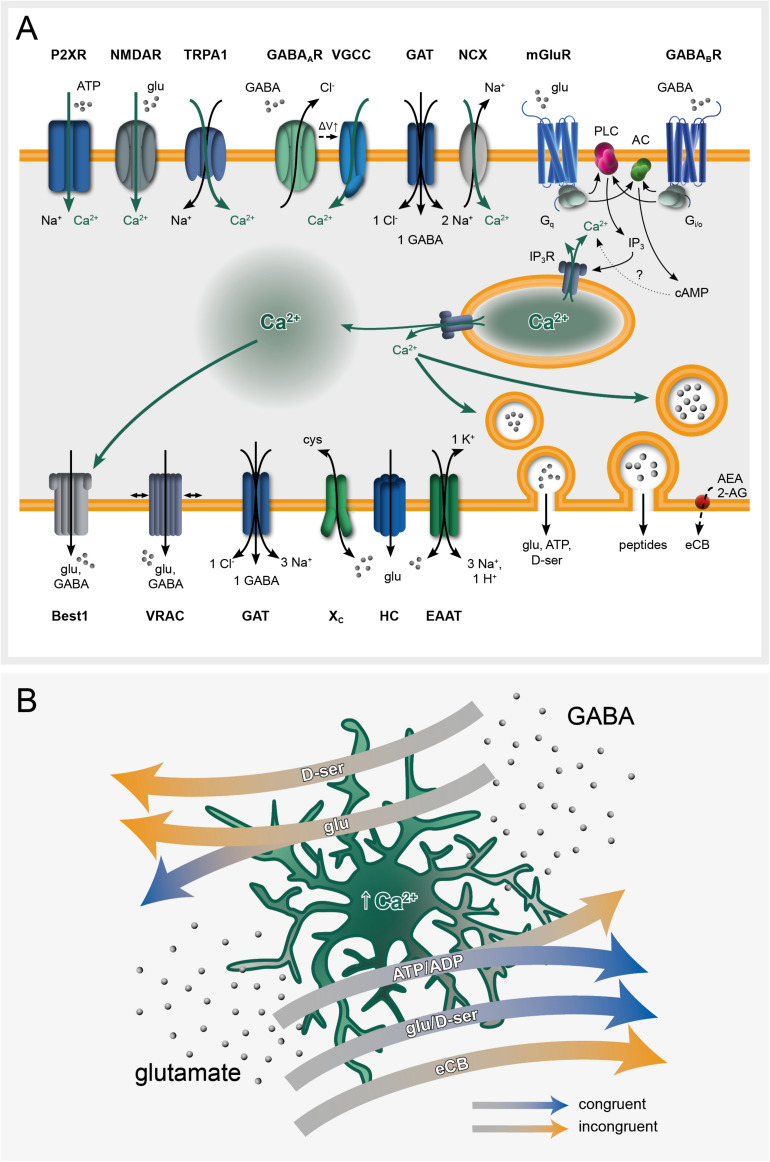
Ca^2+^ signaling at the core of astroglial *black box* operations. **(A)** Astroglial receptome coordinates glutamate and GABA-induced intracellular Ca^2+^ signaling and subsequent gliotransmitter release. **(B)** Glutamate and GABA elicit Ca^2+^-dependent gliotransmitter-mediated congruent and incongruent modulation of network plasticity. Congruent signaling refers to contexts in which GABA and glutamate as initial stimuli exert inhibitory or excitatory effects on the neuronal network, respectively. *Vice versa*, incongruent signaling designates scenarios in which GABA has an excitatory and glutamate an inhibitory final impact on the network. Both congruent and incongruent signaling may involve the same gliotransmitters (highlighted on the arrows). 2-AG, 2-arachidonoylglycerol; AC, adenylate cyclase; ADP/ATP, adenosine di/tri-phosphate; AEA, anandamide; Best1, bestrophin-1 channel; cAMP, cyclic AMP; cys, cysteine; D-ser, D-serine; EAAT, excitatory amino acid transporter; eCB, endocannabinoids; GABA, γ-aminobutyric acid; GABA_A_R/GABA_B_R, GABA receptors; GAT, GABA transporter; glu, glutamate; HC, hemichannel; IP_3_, inositol triphosphate; IP_3_R, inositol triphosphate receptor; mGluR, metabotropic glutamate receptor; NCX, sodium-calcium exchanger; NMDAR, N-methyl-D-aspartate receptor; P2XR, purinergic transmitter-gated ion channels; PLC, phospholipase C; TRPA1, transient receptor potential A1; VGCC, voltage-gated calcium channel; VRAC, volume-regulated anion channel; X_C_, cysteine-glutamate antiporter; ΔV, membrane potential.

The astroglial membrane receptome, responsible for transmitter-triggered Ca^2+^ signaling is highly diverse and is far from being fully characterized (Figure 1A). In the following, we focus on glutamatergic and GABAergic signaling, representing by far the major emblems of the black-and-white E/I dichotomy. Astrocytes can express various types of ionotropic Ca^2+^ permeable glutamate receptors: AMPA receptors in cortex and cerebellum (Schipke et al., 2001; Lalo et al., 2006; Saab et al., 2012) and NMDA receptors in the cortex (Schipke et al., 2001; Lalo et al., 2006; Kirchhoff, 2017), although their expression in the hippocampus is still unclear (Matthias et al., 2003; Verkhratsky and Kirchhoff, 2007; Serrano et al., 2008; Letellier et al., 2016). Kainate receptors may be absent from astroglial membranes under physiological conditions, but were reported to be inducible in a mouse model of temporal lobe epilepsy (TLE; Das et al., 2012; Vargas et al., 2013). Among metabotropic glutamate receptors, G_q_-coupled mGluR5 activation results in IP_3_-mediated Ca^2+^ increase through the phospholipase C pathway (Panatier and Robitaille, 2016). mGluR5 contribution to Ca^2+^ oscillations seems to be restricted, at least in the adult brain, to the fine perisynaptic processes (Sun et al., 2013, 2014; Haustein et al., 2014). Astrocytes express also the G_i/o_-coupled mGluR2/3, whose activation leads to inhibition of adenylate cyclase (Aronica et al., 2003; Sun et al., 2013; Figure 1A).

With respect to GABAergic signaling, astrocytes display internal Ca^2+^ increases following GABA_A_ receptor-mediated depolarization through voltage-gated Ca^2+^ channels (VGCCs; Nilsson et al., 1993; Verkhratsky and Steinhäuser, 2000; Meier et al., 2008; Parpura et al., 2011; Verkhratsky et al., 2012). However, given the low membrane input resistance of mature astrocytes, the contribution of GABA_A_ receptors to Ca^2+^ responses *in vivo* remains controversial. Metabotropic G_i/__o_-coupled GABA_B_ receptors were extensively reported to induce intracellular Ca^2+^ rises in an IP_3_-dependent manner (Mariotti et al., 2016; Nagai et al., 2019) followed by gliotransmitter release (Serrano et al., 2006; Perea et al., 2016; Durkee et al., 2019). Although their role in the cortical astroglial response is unclear, GABA transporter (GAT)-mediated Na^+^ symport increases intracellular Ca^2+^ through the Na^+^/Ca^2+^ exchanger (Doengi et al., 2009; Boddum et al., 2016), as previously suggested for glutamate transporters (Schummers et al., 2008). Synergistic activation of different pathways is likely to occur upon GABAergic signaling, thus potentially introducing an additional level of up-stream signal discrimination (Matos et al., 2018; Figure 1A).

There is plenty of evidence that gliotransmitter release occurs (Parpura et al., 1994; Jeftinija et al., 1997; Bezzi et al., 1998) and that it is, at least partially, a Ca^2+^ dependent mechanism (Bezzi et al., 2004; Perea and Araque, 2005; Araque and Navarrete, 2010; Schwarz et al., 2017; Bohmbach et al., 2018; Savtchouk and Volterra, 2018). Glutamate release occurs through several pathways, including reverse operation of plasma membrane glutamate transporters (Longuemare and Swanson, 1997; Rossi et al., 2000), cystine-glutamate Xc- antiporter (Cavelier and Attwell, 2005), volume-regulated anion channels (VRACs; Kimelberg et al., 1990; Mongin and Kimelberg, 2005; Abdullaev et al., 2006; Liu et al., 2006; Ramos-Mandujano et al., 2007), P2X_7_ receptors (Duan et al., 2003), the Ca^2+^ activated anion channel bestrophin 1 (Best1; Park et al., 2009; Woo et al., 2012), hemichannels (Ye et al., 2003) and vesicular exocytosis (Jeftinija et al., 1997; Bezzi et al., 2004; Montana et al., 2006; Bowser and Khakh, 2007; Xu et al., 2007; Parpura and Zorec, 2010; Schwarz et al., 2017). In contrast to glutamate, GABA release mechanisms were less extensively addressed and remain elusive. Vesicular release of GABA seems unlikely due to the lack of GABA-containing synaptic vesicles in astrocytes (Yoon and Lee, 2014). *Ex vivo* electrophysiological studies using acute brain slices suggested that VRACs (Kozlov et al., 2006; Le Meur et al., 2012) as well as GATs (Barakat and Bordey, 2002; Richerson and Wu, 2003; Lee et al., 2011) can mediate GABA release. GAT2/3 are indeed involved in astroglial GABA release (Héja et al., 2012; Unichenko et al., 2013), as well as Best1 (Lee et al., 2010). Although still under debate, both GATs and Best1 could be responsible for tonic as well as phasic GABAergic astroglial signaling under physiological conditions. Remarkably, reactive astrocytes show aberrant and abundant tonic GABA release through Best1 in mouse models of Alzheimer’s disease (Jo et al., 2014). In a mouse model of TLE, the astrocyte-specific rescue of Best1 could restore tonic GABAergic inhibition and suppressed seizure susceptibility in Best1 complete knock-out mice (Pandit et al., 2020).

## The Paradox of Astroglial *Black Box* Function in the Neural Network

The astrocytes’ *black box* operations typically involve an initial stimulus (most commonly synaptically released neurotransmitters), an astroglial receptor inducing an intracellular signaling cascade leading to Ca^2+^ elevations, a released gliotransmitter and finally the net effect on the neuronal network: excitation or inhibition. Assuming that GABA as initial stimulus would exert an inhibitory (congruent) rather than excitatory (incongruent) and glutamate an excitatory (congruent) rather than inhibitory (incongruent) effect on the network, several scenarios co-exist along this information processing thread (Figure 1B and Table 1).

**TABLE 1:** Summary of congruent and incongruent signaling pathways evoked by glutamate and GABA, according to brain region, released gliotransmitter, and principal neuronal targets.

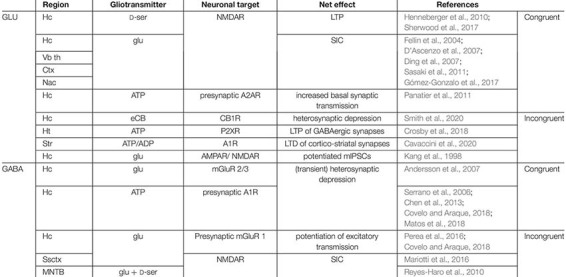

*A1R, adenosine 1 receptor; A2AR, adenosine 2A receptor; ADP/ATP, adenosine di/tri-phosphate; CB1R, cannabinoid receptor 1; Ctx, cortex; D-ser, D-serine; eCB, endocannabinoids; GABA, γ-aminobutyric acid; glu, glutamate; Hc, hippocampus; Ht, hypothalamus; LTD, long-term depression; LTP, long-term potentiation; mGluR, metabotropic glutamate receptor; MNTB, medial nucleus of the trapezoid body; Nac, nucleus accumbens; NMDAR, N-methyl-D-aspartate receptor; SIC, slow inward current; Str, striatum; Ssctx, somatosensory cortex; Vb th, ventro-basal thalamus.*

### Congruent Signaling Preserves Input–Output Polarity

In the simplest case, initial stimulus and net result are congruent as it has been observed frequently across brain regions. Cortical, hippocampal and thalamic astrocytes stimulated by glutamate release glutamate and in turn induce NMDA receptor-dependent slow inward currents (SICs) in adjacent neurons (D’Ascenzo et al., 2007; Ding et al., 2007; Pirttimaki et al., 2011; Sasaki et al., 2011; Gómez-Gonzalo et al., 2017), resulting in neuronal synchronization and an elevated network excitability (Fellin et al., 2004). However, a congruent net result can also be obtained when the released gliotransmitter is neither glutamate nor GABA. Within the hippocampal network, both glutamate and GABA, as neurotransmitters, can stimulate ATP release from astrocytes and thereby evoke congruent consequences for the neuronal environment. Glutamate-induced ATP release enhances basal synaptic transmission at pyramidal cells (Panatier et al., 2011), while GABA-induced ATP release generates (hetero-) synaptic depression (Serrano et al., 2006; Andersson et al., 2007; Chen et al., 2013; Covelo and Araque, 2018) or up-regulation of inhibitory transmission (Matos et al., 2018). Alternatively, glutamate can stimulate astrocytes to release the NMDA receptor co-agonist D-serine, being an essential component of hippocampal long-term potentiation (LTP; Henneberger et al., 2010; Sherwood et al., 2017). Most strikingly, GABAergic stimulation can lead to astroglial release of glutamate, anticipating a reversal of the initial stimulus’ nature, yet still exhibiting a net dampening effect on the hippocampal network through potentiated inhibitory postsynaptic currents (IPSCs; Kang et al., 1998) or heterosynaptic depression (Andersson et al., 2007). Astrocytes therefore can convey the initial message of the primary stimulus to the network, sometimes via alternative routes (ATP, D-serine) and even by switching from an “inhibitory” GABAergic stimulation to glutamate release (Figure 1B and Table 1).

### Incongruent Signaling Reverses Input–Output Polarity

Astrocytes can change the nature of the initial stimulus with respect to the output to the network. The most evident way to achieve this is to perform a switch between the main inhibitory and the main excitatory transmitter. Indeed, GABAergic stimulation of astrocytes can be turned into glutamatergic excitation and thereby potentiate excitatory transmission at the CA3-CA1 microcircuit (Mariotti et al., 2016; Perea et al., 2016; Covelo and Araque, 2018). Likewise, an astroglial switch is attained by including impartial transmitters in the information processing thread. Hypothalamic astrocytes receiving concomitant glutamatergic input and cholecystokinin stimulation release ATP. Acting on P2X-receptors, this astrocyte-derived purinergic stimulation is crucial for LTP of local GABAergic synapses, thereby augmenting the inhibitory tone of the network (Crosby et al., 2018). Moreover, striatal astrocytes also switch a glutamatergic input into long-term depression (LTD) mediated via purines (Cavaccini et al., 2020). Similarly, endocannabinoids released after glutamatergic activation mediate transient hippocampal heterosynaptic depression (Smith et al., 2020). Conversely, GABA-evoked astroglial Ca^2+^-transients induce the co-release of glutamate and D-Serine, thereby producing NMDA receptor-dependent SICs in principal neurons of the medial nucleus of the trapezoid body (MNTB, ascending auditory pathway) (Reyes-Haro et al., 2010). In contrast to hippocampal pyramidal neurons, however, the postsynaptic principal neurons of the MNTB are less susceptible to SIC-mediated synchronization. Moreover, astroglial switches modulating synaptic plasticity operate at various timescales. In contrast to mechanisms such as LTP and (transient) heterosynaptic depression acting within minutes, striatal astrocytes increase the number of excitatory synapses and enhance excitatory transmission upon GABAergic stimulation, prevailing for at least 48 h. Mechanistically, the activation of the G_i_-pathway downstream of the astroglial GABA_B_ receptor leads to an upregulation of the synaptogenic multi-domain matrix glycoprotein thrombospondin-1 *in vivo* (Nagai et al., 2019).

Astrocytes therefore can reverse the initial message of the primary stimulus to the network with the same mechanisms employed to convey a congruent message: by switching from an “inhibitory” GABAergic stimulation to glutamate release, using alternative routes (ATP, D-serine, endocannabinoids), but oppositely even by reproducing the initial stimulus (glutamate-induced glutamate release) (Figure 1B and Table 1). For sake of simplicity, here the concept of congruent and incongruent signaling has been mainly reduced to contexts involving glutamate or GABA as initial stimulus, followed by Ca^2+^-dependent gliotransmitter release. However, gliotransmitters’ impact on neuronal signaling encompasses several additional levels of complexity. Glia-driven purinergic signaling for example can induce down regulation of neuronal GABA_A_ (Lalo et al., 2014) as well as NMDA receptors (Lalo et al., 2016), thereby potentially acting in both congruent and incongruent ways. Furthermore, astroglial Ca^2+^-sensitive Best-1 channels have been shown to release glutamate and GABA, in a region-dependent manner. While hippocampal astrocytes predominantly release glutamate (Woo et al., 2012), putatively due to a lack of GABA content (Yoon et al., 2011), cerebellar astrocytes do contain and release GABA (Lee et al., 2010).

Generalizing the gathered evidence, stimulation of the same astroglial receptor can lead to the release of different gliotransmitters. Stimulation of different receptors can have the same output and the same gliotransmitter can finally have different actions on the network. This paradoxical feature of astrocytes can be partially resolved by dissecting the information processing steps attributed to the astrocyte function itself or the context it is embedded in.

### Astroglial Ca^2+^ Bottleneck: Upstream and Downstream Signal Discrimination and Processing

Astrocyte-mediated neuronal plasticity starts with the detection and discrimination of a stimulus. At the multipartite synapse, astroglial processes frequently interact with GABAergic interneurons and glutamatergic principal neurons. Specifically, cortical as well as hippocampal astroglia have the capacity to engage in interneuron-type specific interactions. Remarkably, they are more responsive to somatostatin compared to parvalbumin interneuron stimulation, based on the co-release of the neuropeptide somatostatin (Mariotti et al., 2018). Subsequently, the selective sensitivity to somatostatin interneurons is integrated and translated into an astroglia-mediated up-regulation of the synaptic inhibition of pyramidal neurons (Matos et al., 2018). Moreover, neuronal activity is also decoded in spatiotemporal patterns of circuit association, firing duration and frequency. Astrocytes are capable of discriminating synaptic activity of different neuronal circuits within the hippocampus, even if the same transmitter is released. This is likely due to distinct patterns of receptor expression and the existence of subcellular functional domains (Perea and Araque, 2005; Shigetomi et al., 2008). In the hippocampus, neuronal firing rates can be correlated with astroglial Ca^2+^ signals, i.e., high frequency synaptic activity depresses and low frequency increases Ca^2+^ signals (Perea and Araque, 2005). Moreover, neuronal firing patterns lead to distinct gliotransmitter release profiles, differentially affecting the neuronal network. While low or brief interneuron stimulation evokes glutamate release, high or sustained interneuron stimulation provokes the co-release of glutamate and purines (Covelo and Araque, 2018).

At the core of the astroglial *black box*, intracellular Ca^2+^-signals remain loosely defined. Even though a variety of Ca^2+^ signals have been mapped according to their spatiotemporal properties, still analysis and interpretation remain a major challenge (Nimmerjahn et al., 2009; Khakh and Sofroniew, 2015; Rusakov, 2015; Bazargani and Attwell, 2016; Bindocci et al., 2017; Stobart et al., 2018; Semyanov et al., 2020). So far, distinct Ca^2+^-signals have not been associated to specific intracellular signaling cascades or the nature of synaptic plasticity. The current consensus, however, states that spatially restrained Ca^2+^ signals will likely induce homosynaptic modulation while larger, propagating Ca^2+^ signals intervene in heterosynaptic and territorial synaptic plasticity (Araque et al., 2014). In particular, the integrative function of astrocytes, i.e., the computation of simultaneously converging signaling pathways in a probably non-linear fashion, remains to be elucidated (Durkee and Araque, 2019). Similarly, only few classes of Ca^2+^ signals could be clearly attributed to a functional correlate such as sensory stimulation (Wang et al., 2009; Stobart et al., 2018) or locomotion and startle responses (Paukert et al., 2014).

Downstream of intracellular Ca^2+^ signal integration, astrocytes can modulate gliotransmitter release and thereby determine the final impact on neuronal plasticity. Recent work supports the concept that the same astrocyte can release different gliotransmitters based on the existence of individual vesicle populations containing different gliotransmitters, operating through distinct v-SNARE proteins and oppositely regulating synaptic plasticity (Schwarz et al., 2017; Covelo and Araque, 2018). A further level of complexity arises from the coexistence of different mechanisms of gliotransmission with specific Ca^2+^-dependency. Vesicular release of gliotransmitters is threshold-based and requires relatively high intracellular Ca^2+^ elevations (Kreft et al., 2003; Parpura and Zorec, 2010). Ca^2+^-dependent opening of large conductance anion channels like Best1 is sigmoidal and has an EC50 for [Ca^2+^]_i_ of about 150 nM (Lee et al., 2010), thus making it a suitable mechanism of gliotransmitter release even at resting conditions or in response to minute Ca^2+^ elevations (Clapham, 2007).

Finally, the net outcome of astroglial *black box* operations depends on the context of targeted neuronal receptors and the connectivity of the local circuitry. Especially in glutamatergic and purinergic transmission, the stimulus nature can be switched if presynaptic rather than postsynaptic receptors are activated (Serrano et al., 2006; Andersson et al., 2007; Hulme et al., 2014; Cavaccini et al., 2020). The connectivity of local circuits plays a major role when inhibitory transmission is potentiated by an excitatory transmitter (Kang et al., 1998; Crosby et al., 2018; Mederos and Perea, 2019), inhibitory transmission is inhibited, or excitatory transmission is disinhibited (Liu et al., 2004; Yarishkin et al., 2015). Associated to specific local circuits, distinct astroglial subpopulations (Zhang and Barres, 2010; Liddelow et al., 2017; Morel et al., 2017) interact with designated neuronal subtypes or circuits such as the striatal dopaminergic D1/D2 pathways (Martín et al., 2015; Martin-Fernandez et al., 2017). Given the kaleidoscopic complexity added to brain and network function, the astroglial syncytium holds a multitude of targets to be explored in health and disease.

## Aberrant Ca^2+^ Signaling and Network Excitability Disruption in Epilepsy

The (im-) balance of E/I is a central element in the pathophysiology of epilepsy. More specifically, large populations of neurons become hyperexcitable and are more likely to engage in synchronous firing episodes. Accordingly, a great proportion of anti-epileptic drugs aim at enhancing GABA-mediated inhibition and decreasing glutamate-mediated excitation (Treiman, 2001; Czapiński et al., 2005). Astrocytes are portrayed as key players in epilepsy due to their diverse roles in the modulation of neuronal excitability and synchrony (Coulter and Steinhäuser, 2015; Robel and Sontheimer, 2016). A central property in this context is Ca^2+^-dependent astroglial glutamate release, driving NMDA receptor-induced neuronal excitation and favoring neuronal synchrony (Parri et al., 2001; Angulo et al., 2004; Fellin et al., 2004; Tian et al., 2005; Poskanzer and Yuste, 2011; Sasaki et al., 2014). Importantly, astroglial Ca^2+^ signals themselves are generally perturbed under neuropathological conditions (Kuchibhotla et al., 2009; Jiang et al., 2016; Mizuno et al., 2018; Shigetomi et al., 2019). Tonic long-lasting Ca^2+^ elevations result at least partially from excitotoxic spilling of glutamate, GABA and ATP from dying cells (Shigetomi et al., 2019).

Astrocyte (dys-) functions have been causally linked to the etiology of TLE (Bedner et al., 2015; Steinhäuser et al., 2016; Deshpande et al., 2020). On a cellular and molecular level, this is paralleled by a selective vulnerability of GABAergic interneurons (Sanon et al., 2005; Upadhya et al., 2019) and reduced glutamate decarboxylase (GAD) as well as glutamine synthetase (GS) activity in astrocytes (Robel and Sontheimer, 2016; Chan et al., 2019), leading to reduced vesicular GABA levels in neurons and subsequently reduced inhibitory input on hippocampal pyramidal neurons (Liang et al., 2006). However, glutamate levels also increase (Luna-Munguia et al., 2011) and a re-emergence of astroglial mGluR5 receptors can be observed (Umpierre et al., 2019). Recent lines of evidence also support a role for purinergic signaling in the etiology and progression of epilepsy and therefore further suggest that astrocytes actively contribute to this pathological scenario as modulators of the ATP/adenosine signaling (Boison, 2016; Beamer et al., 2017; Boison and Steinhäuser, 2018). Astroglial Ca^2+^ signals are pivotal for understanding the pathophysiology of epilepsy (Carmignoto and Haydon, 2012). *Ex vivo*, focal, seizure-like discharge onset and maintenance are associated with substantial astroglial Ca^2+^ transients, generating recurrent excitatory loops with neurons (Gómez-Gonzalo et al., 2010; Álvarez-Ferradas et al., 2015). *In vivo*, astroglial Ca^2+^ signals promote the propagation of epileptiform activity in the hippocampus. In fact, at seizure onset astrocytes display Ca^2+^ elevations prior to neurons and the suppression of those preceeding signals in Itpr2^–^/^–^ mice reduces seizure activity (Heuser et al., 2018). These data suggest a modulatory, pro-epileptic effect of astroglial Ca^2+^ signals (Fellin et al., 2006; Heuser et al., 2018). Finally, seizure termination by spreading depolarization (SD) is accompanied by large Ca^2+^ waves in astrocytes, whose importance has been largely overlooked so far (Seidel et al., 2016; Heuser et al., 2018).

As it is for TLE, both glutamatergic- and GABAergic signaling are intimately involved in the generation and spreading of slow-wave discharges (SWDs) normally occurring in the thalamocortical network during sleep and pathologically in absence epilepsy (Tanaka et al., 1997; Tancredi et al., 2000; Crunelli and Leresche, 2002; Melø et al., 2006; Gould et al., 2014). *In vivo* astroglial Ca^2+^ activity temporally precedes the rhythmic and synchronized neocortical slow oscillations (∼1 Hz) which are typical for sleep. Indeed, optogenetic activation of astrocytes can induce this slow-oscillation state, possibly through glutamate transients (Poskanzer and Yuste, 2016). In line with this, neuronal firing reliably follows astroglial network synchronization during slow-wave activity. The fraction of astrocytes and neurons involved in the synchronous slow-wave state decreases both after astroglial uncoupling and intracellular Ca^2+^ chelation using EGTA (Szabó et al., 2017). Moreover, astroglial Ca^2+^ signaling is involved in the *in vivo* mGluR2-dependent disinhibition of neurons of the thalamic ventro-basal nucleus, thus playing a key role in sensory information processing (Copeland et al., 2017). On the other hand, dampening of astroglial Ca^2+^ oscillations in the striatum was linked to the upregulation of GAT3 expression and the resulting GABA uptake, thus reducing tonic inhibition and exacerbating neuronal excitability (Yu et al., 2018). The role of astrocytic Ca^2+^ transients in network synchronization and pathology is therefore still controversial.

## Conclusion

Neuronal network plasticity can be simplified in terms of E/I, represented by glutamate and GABA, respectively. Including the discriminatory and integrative function of astrocytes into the equation reveals a profound entanglement of E/I, making these traditional labels insufficient. Astrocytes can preserve (*congruence*) or reverse (*incongruence*) neuronal inputs based on the engagement of distinct receptor arrays as well as the integration of converging Ca^2+^ signaling cascades and orchestrated gliotransmitter release. Furthermore, the association of astroglial subpopulations to specific neuronal circuits adds a further layer of complexity. To date, further efforts are required in order to understand astroglial *black box* operations and link them to Ca^2+^ signal heterogeneity. Advances in decoding astroglial Ca^2+^ signaling could reveal the untapped therapeutic potential in pathologies emerging from network excitability dysregulation, as suggested by the collectively acknowledged role of astroglial Ca^2+^ in epilepsy.

## Author Contributions

LC and DG equally contributed to the review, screened the literature, wrote the first draft, conceptualized the table, designed the figure with AS, and finalized the manuscript. AS and FK reviewed and finalized manuscript and figure. All authors approved on the final version of the manuscript.

## Conflict of Interest

The authors declare that the research was conducted in the absence of any commercial or financial relationships that could be construed as a potential conflict of interest.
